# Physicochemical and nutritional properties of extrudates from food grade distiller's dried grains, garbanzo flour, and corn grits

**DOI:** 10.1002/fsn3.769

**Published:** 2018-08-22

**Authors:** Poonam Singha, Sushil K. Singh, Kasiviswanathan Muthukumarappan, Padmanaban Krishnan

**Affiliations:** ^1^ Department of Agricultural & Biosystems Engineering South Dakota State University Brookings South Dakota; ^2^ Department of Dairy and Food Science South Dakota State University Brookings South Dakota

**Keywords:** distiller's dried grains, extrusion, gluten free, protein, total dietary fiber

## Abstract

Distiller's dried grains and garbanzo flour were blended with corn grits for the development of extruded snacks using a single screw extruder. Distiller's dried grains were processed for food application and termed as food grade distiller's dried grains or FDDG. Effects of different level of FDDG addition (0%–20%) and extrusion process parameters such as barrel and die temperature (100–140°C), screw speed (100–200 rpm), and feed moisture content (14%–20% wet basis) on the physical properties (expansion ratio, bulk density, color parameters), functional properties (water absorption and solubility indices), and nutritional properties (total dietary fiber, soluble and insoluble dietary fiber and protein content) of the extrudates were investigated and optimized using response surface methodology. FDDG incorporation had a significant effect (*p* < 0.05) on the total dietary fiber, color parameters, water solubility, and water absorption indices of the extruded snacks. Desirable expanded extrudates with a high level of total dietary fiber and protein were obtained with blends containing 20% FDDG extruded at 140°C extrusion temperature, 167 rpm screw speed, and 19% feed moisture content. Results indicate garbanzo flour, and FDDG can be successfully blended with corn grits to produce nutritious gluten‐free extruded snacks which are high in protein and dietary fiber.

## 1 INTRODUCTION

Modern life is characterized by limited free time and long working hours, which makes it difficult for most people to have proper meals resulting in an increase in consumer preference for ready‐to‐eat products. This lifestyle is now demanding for single‐portion, portable, and healthier snack products (Euromonitor International, 2015). Growing numbers of the young population are attracted to ready‐to‐eat meals and snack products which are not only tasty but also easy to be consumed. According to a report published by Mintel (2015), in the United States, 94% of consumers snack at least once a day, while more than 50% are likely to snack 2–3 times a day. As snacking is replacing traditional meals, 33% of consumers are snacking on healthier foods more than ever before. Hence, the manufacture of ready‐to‐eat products has increased significantly using several types of processing techniques. One such well‐established food processing technique in industries is “extrusion.” Extrusion is a continuous high‐temperature process of cooking, mixing, and forming (Singh & Muthukumarappan, 2017b). Direct expanded food products can be produced using the extrusion process. It has the flexibility in producing different products such as breakfast cereals, cereal baby foods, snack foods, and pasta. A variety of ingredients can be chosen and processed in an extruder. Manufacturers use this process to produce snacks with varied ingredients without the requirement of the frying process, thus making them healthy for consumers. As it is a high‐temperature unit operation, the extrusion process eliminates some of the naturally occurring toxins (Cazzaniga, Basilico, Gonzalez, Torres, & de Greef, 2001; Elias‐Orozco, Castellanos‐Nava, Gaytán‐Martínez, Figueroa‐Cárdenas, & Loarca‐Piña, 2002) making them safer for consumption.

During the extrusion process, the screw exerts shearing action on starch and protein‐based materials and along with high‐temperature transforms the material to a viscoelastic mass. At the exit of the die, the pressure drops suddenly and the emerging material expands (Singha & Muthukumarappan, 2017). The physical properties of the product (density, expansion, texture, etc.) rely on the material composition (such as the presence of protein, starch, fiber, and moisture), and the processing conditions during extrusion.

Cereals and grains usually contain a high percentage of starch (56%–74%). During extrusion cooking, starch undergoes significant structural changes such as gelatinization, melting, and fragmentation. These molecular changes mainly affect the rheological properties of starch which in turn influences the expansion properties of starch during extrusion. This is why most extruded snacks contain cereals and grains. However, extruded snacks prepared only from cereals and grains tend to be low in protein and many other nutrients. Hence, a combination of cereal/grains and legumes are widely explored. Legumes are an excellent source of protein, fiber, and many nutrients such as iron, folates, potassium, magnesium, and vitamins. To cater to the millennials demand of vegan protein, food industries are developing protein isolates from unconventional legume sources. Garbanzo or chickpea has a good nutritional value with almost 20%–28% protein and protein efficiency ratio of 2.64.

Few studies are available that reports use of garbanzo in the development of extruded snacks (Batistuti, Barros, & Arêas, 1991; Geetha, Mishra, & Srivastav, 2012; Meng, Threinen, Hansen, & Driedger, 2010; Milán‐Carrillo, Reyes‐Moreno, Armienta‐Rodelo, Carábez‐Trejo, & Mora‐Escobedo, 2000; Shirani & Ganesharanee, 2009). Researchers (Bhattacharya & Prakash, 1994; Shirani & Ganesharanee, 2009) reported that incorporation of garbanzo into rice flour decreased product expansion. Previous studies on extrusion of blends containing garbanzo dealt with relatively simple raw material compositions. In those studies, effects of extrusion processing on the important aspect of the nutritional properties (protein digestibility, functionality, and antioxidant) and physical attributes (sensory characteristics) of extrudates of pulse‐based flours are not investigated.

Distiller's dried grains (DDG) is a coproduct during the production of ethanol. Its production has increased enormously owing to the increase in demand and production of ethanol. Corn DDG has been extensively used for the development of aqua feed (Singh & Muthukumarappan, 2014a, 2014b, 2017a). Although DDG is a rich source of protein (35%–40%), there are very few reports on its use in human food. This is probably due to the impurities that are left behind during ethanol production. There is a promising opportunity for the food industry to explore the incorporation of DDG into snack products. Few references (Kim, Maga, & Martin, 1989; Shukla, Muthukumarappan, & Julson, 2005) are available in the literature relating to the application of DDG in extruded snacks. Perhaps in combination with legumes, the useful and alternative route for incorporation of DDG will be established.

We hypothesize that high‐protein high‐fiber corn‐based snacks can be produced employing garbanzo flour (GF) and distiller's dried grains processed for food application (FDDG). We also hypothesize that GF and FDDG may enhance the nutritional and physical characteristics of the corn‐based extruded food product. The physicochemical function of the major compounds in the extruded foods is strongly affected by the processing conditions. Therefore, it is essential to develop a process capable of advancing the utilization potential of pulses or pulse‐derived products with enhanced nutritional and physical characteristics and sensory attributes of the processed food.

The objectives of this study were (a) to develop corn extrudates enriched with GF and FDDG; (b) to investigate the physical, functional, and nutritional properties of the extruded snack; and (c) to optimize the extrusion process using response surface methodology.

## 2 MATERIALS AND METHODS

### 2.1 Raw materials and blend preparation

Distiller's dried grains with solubles (DDGS) were provided by Glacial Lakes Energy LLC, Watertown, SD. The DDGS were processed for food application following the method described by Rosentrater and Krishnan (2006) and are termed as food grade distiller's dried grains or FDDG, henceforth. Garbanzo flour (GF) was purchased from a local store in Brookings, SD. Corn grits (CG) was obtained from Bob's Red Mill (Milwaukie, OR). The CG were ground in a mill (Perten Lab Mill 3610) and passed through 40 mesh screens. Standard methods (AOAC International, 2012) were followed for the determination of protein, fat, fiber, ash, and moisture content of FDDG, GF, and CG. The w/w ratios of FDDG, GF, and CG in the blends were 0:40:60, 5:35:60, 10:30:60, 15:25:60, and 20:20:60. The flours were weighed according to the experimental design and mixed in a mixer (KitchenAid Professional 5 Plus, Troy, OH). The calculated amount of water was added during mixing according to the desired moisture content (Table 1) of the final blends. The blends were then kept in sealed polyethylene bags overnight for moisture equilibration.

**Table 1:** Chemical composition of the raw materials used for the extrusion process

Component (g/100 g)	FDDG	GF	CG
Moisture	0.70 ± 0.03	7.75 ± 0.07	11.46 ± 0.01
Protein	35.12 ± 0.11	22.52 ± 0.09	6.0 ± 0.04
Fat	0.53 ± 0.05	5.94 ± 0.11	1.5 ± 0.08
Ash	1.24 ± 0.06	2.7 ± 0.14	2.0 ± 0.16
Nitrogen‐free extract	54.18 ± 0.09	54.63 ± 0.05	78.14 ± 0.07
Total dietary fiber	44.73 ± 0.76	22.3 ± 1.20	0.83 ± 0.49
Soluble	1.4 ± 1.06	3.4 ± 1.47	0.02 ± 1.73
Insoluble	44.33 ± 0.96	18.9 ± 1.08	0.81 ± 1.18

*Notes*

Values in the column are mean ± *SD* (*n* = 3).

CG: corn grits; FDDG: distiller's dried grains processed for food application; GF: garbanzo flour.

### 2.2 Extrusion processing

The blends were randomly extruded using a 19.18 mm (0.755 in.) barrel inner diameter (i.d.), single screw laboratory extruder (Brabender Intelli‐Torque Plasti‐Corder^®^, South Hackensack, NJ). The rated power of the extruder was 7.5‐HP, and range of operating screw speed was from 0 to 225 rpm. The L/D ratio of the extruder was 20:1. Screw having a compression ratio of 1.5:1 was used for the experiment. A 3‐mm die nozzle was used for all the experiment. Extrudates were collected and air‐dried before further analysis.

### 2.3 Evaluation of product properties

#### 2.3.1 Expansion ratio

The expansion ratio (ER) of the extrudates was calculated by dividing the radial diameter of the extrudates with the diameter of the die nozzle. The radial diameter measurement was taken 10 times, and the average was reported.

#### 2.3.2 Bulk density

The bulk density (BD) of the extrudates was determined following the method recommended by USDA (USDA, 1999). In short, it was calculated by taking the ratio of the mass of extrudates and known volume of the container that it filled up. BD was measured using a standard bushel tester (Seedburo Equipment Company, Chicago, IL).

#### 2.3.3 Color parameters and total color change

The color of the extrudates was measured using Minolta Spectrophotometer (CM‐2500d; Minolta Co. Ltd, Japan), and total color difference (Δ*E*) was determined following Singha and Muthukumarappan (2015). The lightness or brightness of the extrudates was indicated by the L* value where 0–100 represents darkness to lightness color. Redness or greenness of the extrudates was indicated by the a*, where a high positive a* value indicates more red color. The b* value indicates the degree of the yellow‐blue color, with a higher positive b* value indicating more yellowness.

#### 2.3.4 Water absorption and water solubility indices

To determine the water absorption index (WAI) and water solubility index (WSI), the extrudate samples were grinded to fine powders; 2.5 g of the powdered samples was suspended in 30 ml DI water in a tarred 50‐ml centrifuge tube and vortexed for 30 s. The suspension was then centrifuged 3,000 × *g* for 10 min. After centrifuge, the supernatant was transferred to a tarred aluminum cups and dried at 135°C for 2 hr to remove the moisture. The WAI and WSI were then calculated as mentioned by Singh and Muthukumarappan (2016).

#### 2.3.5 Total, soluble, and insoluble dietary fibers

The total, soluble, and insoluble dietary fibers of the ingredients and the extrudates were measured by the AOAC approved method 991.43 (AOAC, 1992).

### 2.4 Experimental design and statistical analysis

Response surface methodology was adopted in the design of experimental combinations. The central composite rotatable design consisted of four numerical independent variables. The variables had values of: *X*_1_ (percentage of FDDG) = 0, 5, 10, 15, and 20; *X*_2_ (barrel and die temperature) = 100, 110, 120, 130, and 140°C; *X*_3_ (screw speed) = 100, 125, 150, 175, and 200 rpm; and *X*_4_ (percentage of moisture content) = 14, 15.5, 17, 18.5, and 20 (Table 2). The total number of observations were 27 with three replicates at the design center. The experimental design and the codes for the processing variables have been reported in Table 3. The responses studied were ER, BD, color parameters, and overall color changes, WAI, WSI, and total dietary fiber (TDF). Analysis of variance (ANOVA) (Tables 6–7, 8, 9) was performed to determine the goodness of the fit and the significance of the effects of each factor on the responses. Statistical analysis was conducted using Design Expert 8.0.7.1 (Statease, Minneapolis, MN). Pearson's correlation coefficient (*R*) was also applied to establish specific correlations using SPSS (16.0) statistical software.

**Table 2:** Independent numerical and categorical variables and their levels

Numerical variables	Symbol	Coded variable levels				
		−2	−1	0	1	2
FDDG (%)	*X*_1_	0	5	10	15	20
Temperature (°C)	*X*_2_	100	110	120	130	140
Screw speed (rpm)	*X*_3_	100	125	150	175	200
Moisture content (% wb)	*X*_4_	14.0	15.5	17.0	18.5	20.0

*Note*

FDDG: distiller's dried grains processed for food application; wb: wet basis.

**Table 3:** Experimental design layout

Run	Coded variables				Actual variables			
	*x*_1_	*x*_2_	*x*_3_	*x*_4_	*X*_1_ (%)	*X*_2_ (%)	*X*_3_ (°C)	*X*_4_ (rpm)
1	−1	1	−1	1	5	18.5	110	175
2	1	1	1	1	15	18.5	130	175
3	0	0	0	2	10	17.0	120	200
4	1	1	−1	1	15	18.5	110	175
5	−1	1	−1	−1	5	18.5	110	125
6	0	0	2	0	10	17.0	140	150
7	−1	−1	−1	−1	5	15.5	110	125
8	1	−1	−1	−1	15	15.5	110	125
9	2	0	0	0	20	17.0	120	150
10	0	−2	0	0	10	14.0	120	150
11	1	1	1	−1	15	18.5	130	125
12	0	0	0	−2	10	17.0	120	100
13	−1	−1	1	1	5	15.5	130	175
14	0	2	0	0	10	20.0	120	150
15	0	0	0	0	10	17.0	120	150
16	1	−1	1	−1	15	15.5	130	125
17	0	0	0	0	10	17.0	120	150
18	0	0	−2	0	10	17.0	100	150
19	−1	1	1	−1	5	18.5	130	125
20	1	1	−1	−1	15	18.5	110	125
21	−1	−1	−1	1	5	15.5	110	175
22	−2	0	0	0	0	17.0	120	150
23	1	−1	−1	1	15	15.5	110	175
24	−1	1	1	1	5	18.5	130	175
25	−1	−1	1	−1	5	15.5	130	125
26	1	−1	1	1	15	15.5	130	175
27	0	0	0	0	10	17.0	120	150

## 3 RESULTS AND DISCUSSION

**Figure 1:**
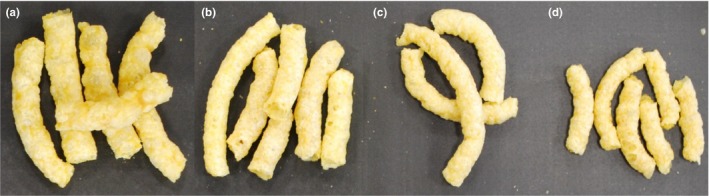
Extruded corn‐based snacks containing (a) 5% FDDG, (b) 10% FDDG, (c) 15% FDDG, and (d) 20% FDDG

The chemical compositions of GF, FDDG, and CG are given in Table 1. The reported values are means of triplicate samples with standard deviations. Figure 1 shows images of some of the extruded snacks.

### 3.1 Expansion ratio

Multiple linear regression equations for ER are shown in Tables 4 and 5. The feed moisture content had a linear effect and FDDG had a quadratic effect on the ER, whereas the screw speed had both the linear and quadratic terms (Table 6).

**Table 4:** Best‐fit response surface models in terms of coded variables after excluding the insignificant terms for ER, BD, L*, a*, b*, Δ*E*, WAI, WSI, and TDF

Parameters	Response surface model	*R* ^2^	*p*‐Value
ER	*Y*_ER_ = 1.81 + 0.21 *x*_3_ − 0.34 *x*_4_ + 0.27 *x*_1_^2^ + 0.17 *x*_3_^2^	0.8314	0.0084
BD (kg/m^3^)	*Y*_BD_ = 212.21 − 41.88 *x*_2_ − 29.42 *x*_3_ + 58.07 *x*_4_	0.7513	<0.0001
L*	*Y*_L*_ = 76.34 − 4.28 *x*_1_ + 2.99 *x*_1_ *x*_2_ + 1.82 *x*_2_ *x*_3_ + 2.13 *x*_1_^2^ − 1.75 *x*_2_^2^	0.8979	0.0006
a*	*Y*_a*_ = 6.77 − 0.7 *x*_3_ + 0.67 *x*_4_	0.4463	0.0089
b*	*Y*_b*_ = 31.47 + 2.67 *x*_3_	0.5336	0.0016
Δ*E*	Δ*E* = 3.80 − 1.61 *x*_3_ + 2.09 *x*_1_ *x*_3_ + 1.94 *x*_2_^2^	0.7727	0.0354
WAI (g/g)	*Y*_WAI_ = 5.64 − 0.46 *x*_3_ − 0.20 *x*_1_ *x*_3_ + 0.17 *x*_1_^2^	0.8614	0.0031
WSI (%)	*Y*_WSI_ = 11.88 + 1.50 *x*_3_ − 0.64 *x*_1_^2^	0.8192	0.0119
TDF (%)	*Y*_TDF_ = 8.19 + 1.46 *x*_1_	0.9605	<0.0001

*Note*

∆*E*: total color difference; a*: red‐green color; b*: yellow‐blue color; BD: bulk density; ER: expansion ratio; L*: lightness; TDF: total dietary fiber; WAI: water absorption index; WSI: water solubility index.

**Table 5:** Best‐fit response surface models in terms of actual variables after excluding the insignificant terms for ER, BD, L*, a*, b*, Δ*E*, WAI, WSI, and TDF

Parameters	Response surface model	*R* ^2^	*p*‐Value
ER	*Y*_ER_ = 53.60 − 0.13 *X*_3_ − 2.84 *X*_4_ + 0.01 *X*_1_^2^ + 0.0003 *X*_3_^2^	0.8314	0.0084
BD (kg/m^3^)	*Y*_BD_ = 257.41 − 4.19 *X*_2_ − 1.18 *X*_3_ + 38.71 *X*_4_	0.7513	<0.0001
L*	*Y*_L*_ = −77.69 − 4.88 *X*_1_ + 0.06 *X*_1_ *X*_2_ + 0.007 *X*_2_ *X*_3_ + 0.085 *X*_1_^2^ − 0.017 *X*_2_^2^	0.8979	0.0006
a*	*Y*_a*_ = 7.08 − 0.03 *X*_3_ + 0.45 *X*_4_	0.4463	0.0089
b*	*Y*_b*_ = 20.56 + 0.11 *X*_3_	0.5336	0.0016
Δ*E*	Δ*E* = 236.32 − 0.10 *X*_3_ + 0.02 *X*_1_ *X*_3_ + 0.02 *X*_2_^2^	0.7727	0.0354
WAI (g/g)	*Y*_WAI_ = 14.18 − 0.14 *X*_3_ − 0.0016 *X*_1_ *X*_3_ + 0.0067 *X*_1_^2^	0.8614	0.0031
WSI (%)	*Y*_WSI_ = 60.23 + 0.285 *X*_3_ − 0.025 *X*_1_^2^	0.8192	0.0119
TDF (%)	*Y*_TDF_ = 4.84 + 0.29 *X*_1_	0.9605	<0.0001

*Note*

∆*E*: total color difference; a*: red‐green color; b*: yellow‐blue color; BD: bulk density; ER: expansion ratio; L*: lightness; TDF: total dietary fiber; WAI: water absorption index; WSI: water solubility index.

**Table 6:** Analysis of variance for expansion ratio, water absorption index, and water solubility index

Source	*df*	Expansion ratio				Water absorption index				Water solubility index			
		SS	MS	*F*‐value	*p*‐Value	SS	MS	*F*‐value	*p*‐Value	SS	MS	*F*‐value	*p*‐Value
Model	14	6.82	0.49	4.23	0.0084	7.96	0.57	5.33	0.0031	86.81	6.20	3.88	0.0119
*X*_1_ *—*FDDG	1	0.20	0.20	1.69	0.2174	0.18	0.18	1.67	0.2205	1.24	1.24	0.77	0.3960
*X*_2_ *—*Temperature	1	0.53	0.53	4.56	0.0541	0.11	0.11	1.01	0.3339	0.50	0.50	0.31	0.5877
*X*_3_ *—*Screw speed	1	1.10	1.10	9.52	0.0095	5.18	5.18	48.52	<0.0001	54.16	54.16	33.93	<0.0001
*X*_4_ *—*Moisture	1	2.71	2.71	23.49	0.0004	0.02	0.02	0.14	0.7117	0.26	0.26	0.16	0.6936
*X*_1_ *X*_2_	1	0.11	0.11	0.91	0.3578	0.04	0.04	0.35	0.5672	1.22	1.22	0.76	0.3998
*X*_1_ *X*_3_	1	0.03	0.03	0.24	0.6359	0.64	0.64	5.96	0.0311	6.80	6.80	4.26	0.0614
*X*_1_ *X*_4_	1	0.33	0.33	2.87	0.1161	0.09	0.09	0.80	0.3888	0.86	0.86	0.54	0.4772
*X*_2_ *X*_3_	1	0.07	0.07	0.63	0.4442	0.37	0.37	3.45	0.0878	1.39	1.39	0.87	0.3699
*X*_2_ *X*_4_	1	0.01	0.01	0.07	0.7994	0.05	0.05	0.42	0.5270	0.03	0.03	0.02	0.8934
*X*_3_ *X*_4_	1	0.06	0.06	0.49	0.4966	0.01	0.01	0.09	0.7713	0.01	0.01	0.00	0.9549
*X*_1_^2^	1	1.51	1.51	13.14	0.0035	0.59	0.59	5.55	0.0363	8.61	8.61	5.40	0.0386
*X*_2_^2^	1	0.20	0.20	1.72	0.2144	0.02	0.02	0.21	0.6585	0.14	0.14	0.09	0.7691
*X*_3_^2^	1	0.62	0.62	5.36	0.0391	0.23	0.23	2.12	0.1712	1.52	1.52	0.95	0.3489
*X*_4_^2^	1	0.38	0.38	3.28	0.0953	0.12	0.12	1.10	0.3148	3.86	3.86	2.42	0.1460
Residual	12	1.38	0.12	—	—	1.28	0.11	—	—	19.15	1.60	—	—
Lack of fit	10	1.02	0.10	0.56	0.7849	0.83	0.08	0.37	0.8830	11.63	1.16	0.31	0.9175
Pure error	2	0.37	0.18	—	—	0.45	0.22	—	—	7.53	3.76	—	—

*Note*

*df*: degrees of freedom; FDDG: distiller's dried grains processed for food application; MS: mean squares; SS: sum of squares.

**Figure 2:**
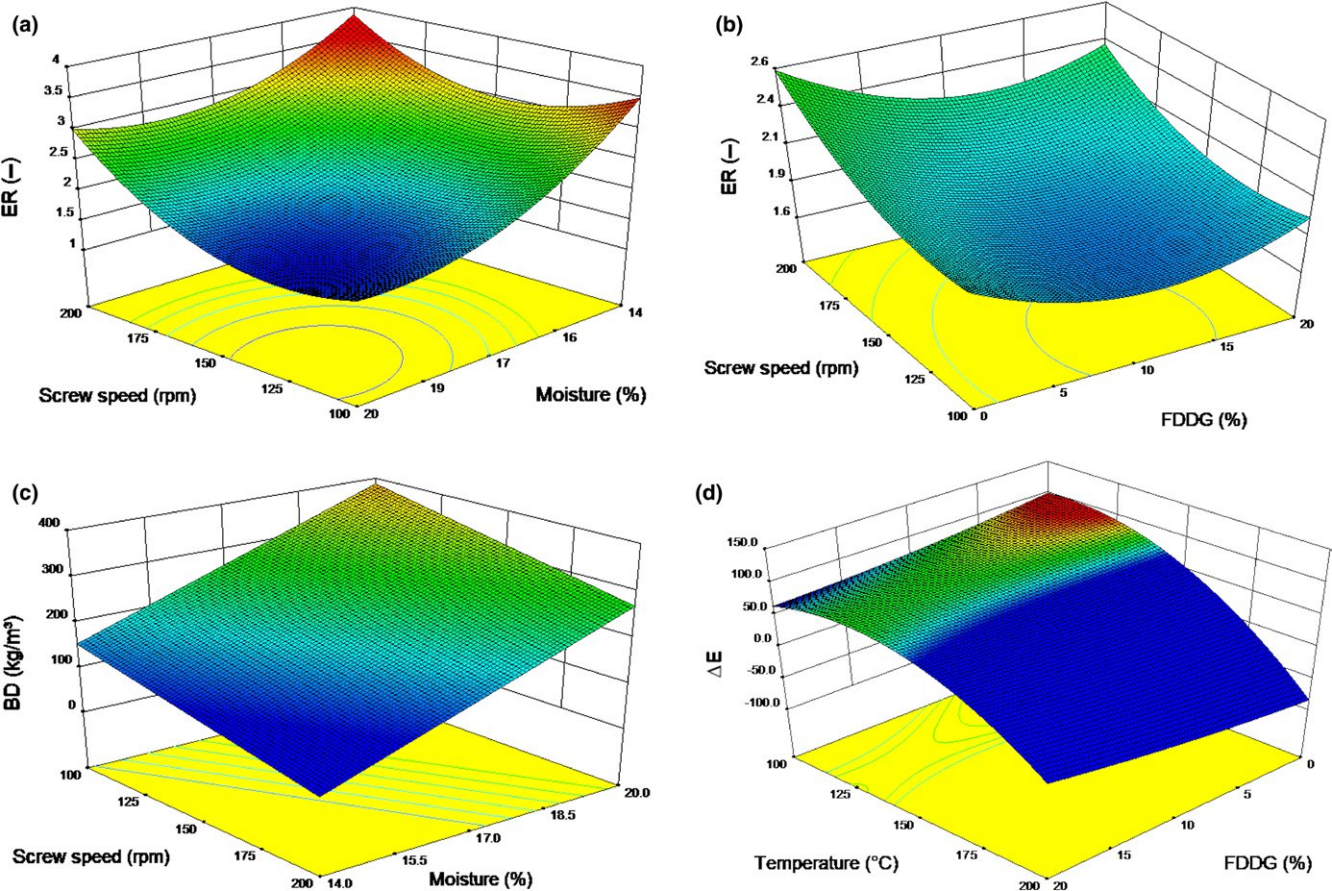
Response surface graphs illustrating the effects of (a) screw speed and moisture on the ER, (b) screw speed and FDDG on the ER, (c) temperature and moisture content on the BD, and (d) temperature and FDDG on the color change of the extruded snacks

The ER of the extruded snacks ranged between 1.45 and 3.56. The highest value of ER was obtained with a FDDG:GF ratio of 5:35 at 15.5% moisture content, 130°C temperature, and 175 rpm screw speed, whereas the lowest value was with 10:30 at 17% moisture content, 120°C temperature, and 150 rpm screw speed. As illustrated in Figure 2a, the ER was high at low moisture content and decreased as the moisture content in the blends increased. According to Oke, Awonorin, Sanni, Asiedu, and Aiyedun (2012), drag force increases when moisture content decreases; as a result, there is more pressure at the die which leads to greater expansion of the extrudates at the exit. The increase in expansion at higher screw speed can be attributed to the shearing effect of the screw (Singha & Muthukumarappan, 2018), which causes protein molecules to be stretched farther apart, weakening bonds and resulting in a puffer product (Filli, Nkama, Jideani, & Ibok, 2012). In addition to that, the shearing effect causes the starch to gelatinize which favored expansion (Chinnaswamy & Hanna, 1988).

Initially, with an increase in the level of the FDDG in the blends, the ER of the extrudates decreased (Figure 2b). This could be due to the overall increase in the protein to the starch ratio in the blends which also affect the starch gelatinization. On further increase in the FDDG level (beyond 10%), there was an increase in the ER. This increase was more pronounced at higher screw speed (more than 175 rpm). The extrudate expansion is also governed by the dough viscosity and elasticity. In our previous study (Singha, Muthukumarappan, & Krishnan, 2018), we have reported that apparent viscosity increased with increase in the FDDG level in the blends during extrusion. This increased viscosity led to lowering rate of contraction on cooling and more mechanical energy into the melt (Singh, 2016). These favor the expansion process as they do not allow the cellular matrix to collapse under high vapor pressure (Ilo, Tomschik, Berghofer, & Mundigler, 1996; Moraru & Kokini, 2003). Thus, lower density products with increased bubble growth are produced.

### 3.2 Bulk density

Bulk density is an important property of the extrudates. BD is commonly influenced by the expansion property that occurs upon exiting of the extrudates from die section (Brown, Fallahi, Muthukumarappan, Singha, & Sindelar, 2015). Multiple linear regression equations for BD are shown in Tables 4 and 5. Table 7 shows the ANOVA of the response function BD in terms of the coded variables. The first‐order terms of temperature, screw speed, and moisture content were significant (*p* < 0.05).

**Table 7:** Analysis of variance for bulk density and total dietary fiber

Source	*df*	Bulk density				Total dietary fiber			
		SS	MS	*F*‐value	*p*‐Value	SS	MS	*F*‐value	*p*‐Value
Model	4	147,323.04	36,830.76	16.62	<0.0001	51.05	12.76	133.77	<0.0001
*X*_1_ *—*FDDG	1	3,534.26	3,534.26	1.59	0.2199	51.01	51.01	534.71	<0.0001
*X*_2_ *—*Temperature	1	42,085.85	42,085.85	18.99	0.0003	0.02	0.02	0.19	0.6665
*X*_3_ *—*Screw speed	1	20,776.51	20,776.51	9.37	0.0057	0.01	0.01	0.10	0.7606
*X*_4_ *—*Moisture	1	80,926.41	80,926.41	36.51	<0.0001	0.01	0.01	0.10	0.7556
Residual	22	48,765.95	2,216.63	—	—	2.10	0.10	—	—
Lack of fit	20	42,020.59	2,101.03	0.62	0.7743	2.09	0.10	17.89	0.0542
Pure error	2	6,745.36	3,372.68	—	—	0.01	0.01	—	—

*Note*

*df*: degrees of freedom; FDDG: distiller's dried grains processed for food application; MS: mean squares; SS: sum of squares.

The BD of the extruded snacks ranged from 98.3 to 445.5 kg/m^3^. The highest value of BD was obtained with FDDG:GF ratio of 5:35 at 18.5% moisture content and extruded at 110°C temperature and at 125 rpm screw speed, whereas the lowest value was with 15:25 at 15.5% moisture content and extruded at 130°C temperature and at 175 rpm screw speed. The BD of the extrudates decreased when the screw speed and the temperature were increased, whereas it increased as the feed moisture content was increased. Such a phenomenon has also been reported by Meng et al. (2010). The decrease in the BD with an increase in extrusion temperature was also observed during extrusion of blends of rice and chickpea flours (Bhattacharya & Prakash, 1994). The degree of superheating of water increases as the extrusion temperature is increased which favors the formation of bubbles. This will result in a decrease in melt viscosity as reported in our previous studies (Singha, 2017; Singha et al., 2018) leading to reduced density. Response surface plot in Figure 2c shows the effect of screw speed and moisture content on the BD. At higher screw speed, lower BD of extrudates was recorded. High screw speeds generate high shear and could have lowered the melt viscosity (Fletcher, Richmond, & Smith, 1985), which favors greater dough elasticity and hence results in a reduced density of the extrudates. Lee and McCarthy (1996) observed increased expansion and reduced BD of rice extrudates as the screw speed was increased. We also observed that with an increase in feed moisture content, BD of the corn‐based extrudates increased. During extrusion of rice‐based snacks, Ding, Ainsworth, Tucker, and Marson (2005) found that feed moisture was the main factor that affected the expansion properties of the extrudates.

### 3.3 Color parameters and total color change

Color is an important sensory attribute of any food product. It provides important information about the extent of thermal treatment during extrusion processing (Chen, Serafin, Pandya, & Daun, 1991). Multiple linear regression equations for color parameters (L*, a*, and b* values) and total color change (Δ*E*) are shown in Tables 4 and 5.

The ANOVA for the L* is shown in Table 8. FDDG had significant linear and quadratic (*p* < 0.05) effects, and temperature had a significant quadratic effect (*p* < 0.05) on the L* values. Interaction effects between FDDG and temperature, and temperature and screw speed were also found to be significant (*p* < 0.05), as confirmed in the ANOVA (Table 8). The lightness (L*) values varied from 64.69% to 91.25%. The L* value decreased with increase in FDDG content in the blends. At high processing temperature, reducing sugars and proteins (amino acids) in food can react and promote nonenzymatic browning, also known as Maillard reaction, which darkens the final product. CG are high in sugars (Wang & Ryu, 2013), and both GF and FDDG are high in protein (amino acids). The observed decrease in L* may be attributed to the Maillard reaction. Reduction in whiteness, as evidenced by the decrease in L* values, indicates darker samples.

**Table 8:** Analysis of variance for lightness (L*) and color change (Δ*E*)

Source	*df*	L*				Δ*E*			
		SS	MS	*F*‐value	*p*‐Value	SS	MS	*F*‐value	*p*‐Value
Model	14	1,025.77	73.27	7.54	0.0006	339.45	24.25	2.91	0.0354
*X*_1_ *—*FDDG	1	440.50	440.50	45.32	<0.0001	16.78	16.78	2.02	0.1810
*X*_2_ *—*Temperature	1	10.80	10.80	1.11	0.3126	0.28	0.28	0.03	0.8586
*X*_3_ *—*Screw speed	1	2.61	2.61	0.27	0.6135	62.51	62.51	7.51	0.0179
*X*_4_ *—*Moisture	1	43.42	43.42	4.47	0.0562	30.82	30.82	3.70	0.0783
*X*_1_ *X*_2_	1	143.76	143.76	14.79	0.0023	24.34	24.34	2.93	0.1129
*X*_1_ *X*_3_	1	20.88	20.88	2.15	0.1684	70.07	70.07	8.42	0.0133
*X*_1_ *X*_4_	1	38.38	38.38	3.95	0.0702	9.67	9.67	1.16	0.3022
*X*_2_ *X*_3_	1	52.71	52.71	5.42	0.0382	0.38	0.38	0.05	0.8346
*X*_2_ *X*_4_	1	22.52	22.52	2.32	0.1539	0.00	0.00	0.00	0.9883
*X*_3_ *X*_4_	1	0.30	0.30	0.03	0.8641	29.05	29.05	3.49	0.0863
*X*_1_^2^	1	97.11	97.11	9.99	0.0082	21.77	21.77	2.62	0.1317
*X*_2_^2^	1	65.26	65.26	6.71	0.0236	80.27	80.27	9.65	0.0091
*X*_3_^2^	1	0.00	0.00	0.00	0.9882	28.45	28.45	3.42	0.0892
*X*_4_^2^	1	7.58	7.58	0.78	0.3946	1.74	1.74	0.21	0.6559
Residual	12	116.63	9.72	—	—	99.86	8.32	—	—
Lack of fit	10	113.97	11.40	8.56	0.1091	98.25	9.83	12.21	0.0780
Pure error	2	2.66	1.33	—	—	1.61	0.80	—	—

*Note*

*df*: degrees of freedom; FDDG: distiller's dried grains processed for food application; MS: mean squares; SS: sum of squares.

The ANOVA for a* (redness) is shown in Table 9. Redness “a*” of the extruded samples ranged from 4.05 to 6.57. Screw speed and moisture content significantly (*p* < 0.05) affected the redness value (Table 9). Redness increased as the moisture content increased, which confirms that whiteness decreased. Redness in food relates to loss of whiteness (Iwe, van Zuilichem, & Ngoddy, 2000).

**Table 9:** Analysis of variance for redness (a*) and yellowness (b*) values

Source	*df*	a*				b*			
		SS	MS	*F*‐value	*p*‐Value	SS	MS	*F*‐value	*p*‐Value
Model	4	26.39	6.60	4.43	0.0089	185.91	46.48	6.29	0.0016
*X*_1_ *—*FDDG	1	2.20	2.20	1.48	0.2373	8.39	8.39	1.14	0.2980
*X*_2_ *—*Temperature	1	1.68	1.68	1.13	0.2993	0.00	0.00	0.00	0.9840
*X*_3_ *—*Screw speed	1	11.61	11.61	7.80	0.0106	170.51	170.51	23.09	<0.0001
*X*_4_ *—*Moisture	1	10.90	10.90	7.32	0.0129	7.01	7.01	0.95	0.3405
Residual	22	32.74	1.49	—	—	162.47	7.39	—	—
Lack of fit	20	32.36	1.62	8.50	0.1104	150.21	7.51	1.23	0.5436
Pure error	2	0.38	0.19	—	—	12.26	6.13	—	—

*Note*

*df*: degrees of freedom; FDDG: distiller's dried grains processed for food application; MS: mean squares; SS: sum of squares.

Yellowness values (b*) of extruded samples ranged from 32.79 to 41.88. The ANOVA for b* is shown in Table 9. Screw speed had a significant effect on yellowness. Yellowness increased as the screw speed increased. Shearing effect increases with increase in the screw speed, and this may favor formation of colored compounds.

The color difference (*∆E*) was used to represent the color change between the blends and the extrudates. The ANOVA for Δ*E* is shown in Table 8. Screw speed had a significant linear effect (*p* < 0.05), and temperature had a significant quadratic effect (*p* < 0.05) on the color difference. The response surface plot for color changes with temperature and FDDG is represented in Figure 2d. As snack products containing GF, FDDG, and CG are not common in the marketplace, there might not be a standard value for the color development of an acceptable snack made from these ingredients. The color data are an important value for future product development of snacks containing GF, FDDG, and CG.

### 3.4 Water absorption index

The WAI is often used as an indication of the degree of starch gelatinization (Ding et al., 2005). It is a measurement of the volume of the swelled starch in excess water and also the integrity of starch in water (Mason & Hoseney, 1986). Multiple linear regression equations for WAI are shown in Tables 4 and 5. Table 6 shows the ANOVA of the response function WAI in terms of the coded variables. From the WAI model, it can be seen that the first‐order term of screw speed and the second‐order term of FDDG were significant (*p* < 0.05). An interaction effect of FDDG and screw speed was also found to be significant (*p* < 0.05).

**Figure 3:**
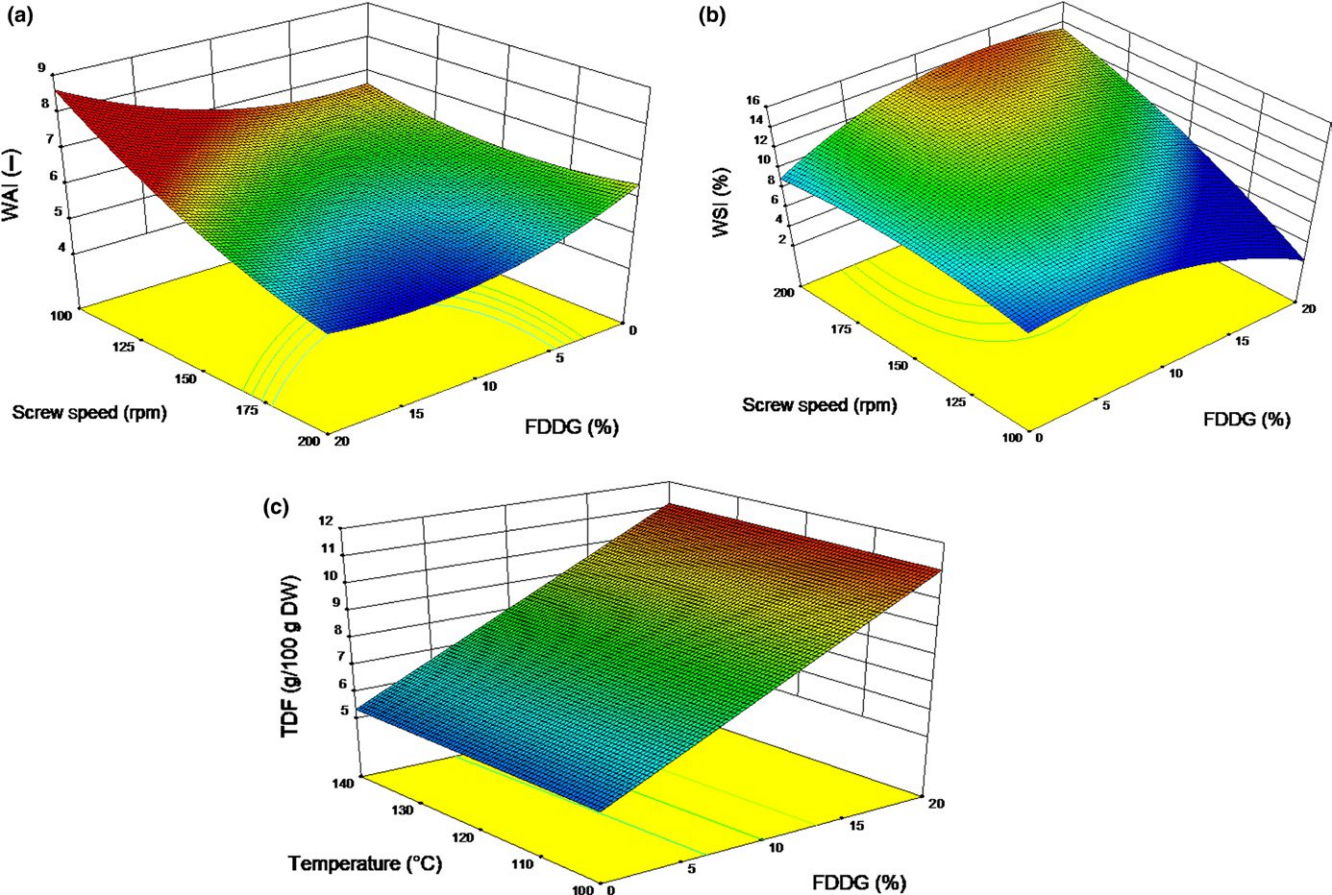
Response surface graphs illustrating the effects of (a) screw speed and FDDG on the water absorption index, (b) screw speed and FDDG on the water solubility index, and (c) FDDG on the TDF of the extruded snacks

In this study, the WAI was in the range of 5.02 and 6.97 g/g dry solid. The effect of GF: FDDG and screw speed on WAI is shown in Figure 3a. It was interpreted that there is a decrease in WAI at high screw speed. At low screw speed with an increase in FDDG level, the WAI increased. Such a phenomenon could be due to the higher water absorption capability of the fiber present in FDDG. A decrease in WAI with an increase in screw speed has been reported in the literature during extrusion of soy white flakes‐based blends (Singh & Muthukumarappan, 2016). At the high shear rate, the availability of undamaged polymers reduces. Hence, there is less availability of hydrophilic groups capable of binding with water molecules. Thus, lower values of WAI are observed at higher extrusion screw speed.

### 3.5 Water solubility index

Due to the extrusion process, there are changes in the physical and chemical nature of the feed ingredients comprising protein, starch, and fiber. The soluble component that is released from starch after extrusion can be measured and is termed as the WSI (Ding, Ainsworth, Plunkett, Tucker, & Marson, 2006) and is often used as an indicator of degradation of molecular components. Multiple linear regression equations for WSI are shown in Tables 4 and 5. Table 6 shows the ANOVA of the response function WSI in terms of the coded variables. The coefficient estimates of WSI model showed that the first‐order term of screw speed and the second‐order term of FDDG were significant (*p* < 0.05).

The WSI ranged from 7.85 to 14.75 with the adequate precision of 6.381 and *R* ^2^ of 0.8192. Maximum WSI was recorded at a moisture content of 18.5%, temperature of 130°C, and screw speed of 175 rpm. The WSI value significantly (*p* < 0.05) increased with increase in the extrusion speed and at the very low level of FDDG (Figure 3b). With the increase in the screw speed, specific mechanical energy increased and this high mechanical shear damaged the macromolecules. As a result, the molecular weight of the starch granules decreased which improved the starch solubility in water (Smith, 1992). In a study by Mezreb, Goullieux, Ralainirina, and Queneudec (2003), WSI was found to increase significantly with increase in screw speed from 200 to 300 rpm for wheat extrudates and from 300 to 500 rpm for corn extrudates.

### 3.6 Total, soluble, and insoluble dietary fibers

Multiple linear regression equations for the TDF are shown in Tables 4 and 5. ANOVA in Table 7 confirms that the model is significant (*p* < 0.001) with insignificant lack of fit (*p* > 0.05). Good coefficient of determination (*R* ^2^ = 0.9605) was obtained with an adequate precision of 43.874. The coefficient estimates of the TDF model showed that only the first‐order term of FDDG was significant (*p* < 0.05).

The TDF ranged between 4.53 and 11.24 (g/100 g DW) in the extruded snacks. The response surface plot in Figure 3c depicts the variation in TDF with changes in FDDG content and temperature. The TDF content of GF and FDDG was 19.75 g/100 g and 44.73 g/100 g, respectively. Hence, the contribution of TDF from FDDG was more than GF. Extrudates with a higher level of FDDG had a higher percentage of TDF content. The FDDG level also had a significant effect (*p* < 0.05) on the SDF and IDF (results not shown). An increase in SDF in the extruded products was observed compared to those in the raw blends. The SDF in the raw blends ranged between 0.97 and 1.37 g/100 g DW and in the extruded products ranged between 2.06 and 5.54 g/100 g DW. On the other hand, there was a slight decrease in the IDF of the extruded products. The IDF in the raw blends ranged from 3.3 to 11.6 g/100 g DW, while in the extruded products, it ranged between 1.78 and 5.7 g/100 g DW.

### 3.7 Relationship between product properties

The degree of puffing of the extrudates as it exits the die nozzle can be estimated from the ER and BD values. ER indicates the expansion only in the perpendicular direction of the extrudate flow, whereas BD is affected by the expansion in all directions (Falcone & Phillips, 1988). The degree of expansion affects the density, fragility, and texture of extrudates (Anton, Gary Fulcher, & Arntfield, 2009; Gujska & Khan, 1991). In our study, a negative correlation (*r* = −0.794, *p* < 0.01) was found between ER and BD. The negative correlation between ER and BD has been reported by many researchers during extrusion cooking (Chevanan, Rosentrater, & Muthukumarappan, 2007). The WAI and WSI were found to have a strong negative correlation (*r* = −0.936, *p* < 0.01). A negative correlation was found between TDF and L* (*r* = −0.622, *p* < 0.01). Our results showed that with an increase in the FDDG level in the extruded products the lightness (L*) value decreased, whereas the TDF of the extrudates increased. The total color change had a negative correlation (*r* = −0.697, *p* < 0.01) with yellowness, whereas a positive correlation (*r* = 0.544, *p* < 0.01) was found with the redness value.

### 3.8 Optimization and model verification

The independent variables were optimized numerically using Design Expert v8. FDDG level was kept maximum, while the remaining independent variables were kept in range. Target goals were assigned for the responses. The WAI and WSI were kept in range, while the TDF and the ER were at a maximum. The optimization resulted in 55 solutions, and the top 10 solutions are shown in Table 10. Validation of the predicted responses was carried out by extruding the blends at three different optimum conditions as shown in Table 10. To check the variability of the predicted responses, two‐tailed, one sample *t* test was carried out. Good agreement was found between the predicted and the experimental values. Results of the *t* test demonstrated no significant difference between the values of recorded responses and the predicted responses. The experimental values for solution 2 were very close to the predicted values. The optimum condition was 20% FDDG, 140°C barrel temperature, 167 rpm screw speed, and 19% moisture content. The extruded snacks produced at the optimum condition had a TDF content of 11.04 g/100 g DW and a crude protein content of 15.91%. A serving of 30 g of the extruded snacks will provide 4.77 g protein, 3.31 g TDF, and 0.084 g of total fat.

**Table 10:** Solutions for optimum conditions and validation

Solution no.	FDDG (%)	Temperature (°C)	Screw speed (rpm)	Moisture (% wb)	ER (—)	WAI (—)	WSI (%)	TDF (g/100 DW)
1	20.00	139.92	152.16	19.91	3.57	5.57	13.24	11.20
2	20.00	139.67	166.50	18.98	3.56	5.51	13.23	11.18
3	20.00	134.55	174.44	20.00	3.88	5.20	14.65	11.17
4	20.00	140.00	183.35	19.31	4.14	5.23	14.75	11.17
5	20.00	138.60	171.78	18.66	3.56	5.49	13.21	11.17
6	20.00	139.93	181.48	18.51	3.85	5.37	13.82	11.16
7	20.00	137.57	192.05	19.00	4.22	5.20	14.75	11.15
8	20.00	130.46	184.11	19.76	3.92	5.10	14.74	11.15
9	20.00	137.35	119.60	14.11	3.56	6.91	7.85	11.14
10	20.00	137.98	121.15	14.00	3.60	6.81	8.20	11.14
Validation								
2	20.00	139.67	166.50	18.98	3.46	5.28	14.74	11.04
3	20.00	134.55	174.44	20.00	3.43	5.27	14.75	10.77
8	20.00	130.46	184.11	19.76	3.54	6.33	9.65	11.03

*Note*

ER: expansion ratio; FDDG: distiller's dried grains processed for food application; TDF: total dietary fiber; WAI: water absorption index; WSI: water solubility index.

## 4 CONCLUSIONS

A corn‐based extruded product containing a varying percentage of FDDG and GF has been produced. Product properties studied were ER, BD, water solubility and absorption indices, color parameters and total color change, and TDF. FDDG level in the blends had a significant effect on the TDF, ER, lightness value, and water solubility and absorption indices. Extrusion screw speed and moisture content of the blends had a significant effect on the ER. Strong negative correlation was found between ER and BD, and between water absorption and water solubility indices. Optimization of the processing conditions to achieve high ER and TDF content in the extruded snacks was carried out. The optimized conditions of independent variables found were FDDG (20%), extrusion temperature (~140°C), screw speed (~166 rpm), and feed moisture content (~19%). Our study shows that some physicochemical and nutritional properties of the extruded snacks could be significantly influenced by raw materials and extrusion processing conditions. Nutritious high‐protein high‐fiber extruded snacks were developed. This study provides a new research on the physicochemical properties of corn‐based extruded snacks containing FDDG and GF.

## CONFLICT OF INTEREST

None declared.
